# Population pharmacogenomics: an update on ethnogeographic differences and opportunities for precision public health

**DOI:** 10.1007/s00439-021-02385-x

**Published:** 2021-10-15

**Authors:** Yitian Zhou, Volker M. Lauschke

**Affiliations:** 1 Department of Physiology and Pharmacology, Karolinska Institutet, 171 77 Stockholm, Sweden; 2 Dr. Margarete Fischer-Bosch Institute of Clinical Pharmacology, Stuttgart, Germany

## Abstract

Both safety and efficacy of medical treatment can vary depending on the ethnogeographic background of the patient. One of the reasons underlying this variability is differences in pharmacogenetic polymorphisms in genes involved in drug disposition, as well as in drug targets. Knowledge and appreciation of these differences is thus essential to optimize population-stratified care. Here, we provide an extensive updated analysis of population pharmacogenomics in ten pharmacokinetic genes (*CYP2D6*, *CYP2C19*, *DPYD*, *TPMT*, *NUDT15* and *SLC22A1*), drug targets (*CFTR*) and genes involved in drug hypersensitivity (*HLA-A*, *HLA-B*) or drug-induced acute hemolytic anemia (*G6PD*). Combined, polymorphisms in the analyzed genes affect the pharmacology, efficacy or safety of 141 different drugs and therapeutic regimens. The data reveal pronounced differences in the genetic landscape, complexity and variant frequencies between ethnogeographic groups. Reduced function alleles of *CYP2D6*, *SLC22A1* and *CFTR* were most prevalent in individuals of European descent, whereas *DPYD* and *TPMT* deficiencies were most common in Sub-Saharan Africa. Oceanian populations showed the highest frequencies of *CYP2C19* loss-of-function alleles while their inferred CYP2D6 activity was among the highest worldwide. Frequencies of HLA-B*15:02 and HLA-B*58:01 were highest across Asia, which has important implications for the risk of severe cutaneous adverse reactions upon treatment with carbamazepine and allopurinol. *G6PD* deficiencies were most frequent in Africa, the Middle East and Southeast Asia with pronounced differences in variant composition. These variability data provide an important resource to inform cost-effectiveness modeling and guide population-specific genotyping strategies with the goal of optimizing the implementation of precision public health.

**Supplementary Information:**

The online version contains supplementary material available at 10.1007/s00439-021-02385-x.

## Introduction

Interindividual differences in drug response are a common phenomenon in pharmacological therapy. While some patients respond appropriately to a given treatment, in others, it can result in lack of efficacy, which affects an estimated 10–45% of patients (Salvà Lacombe et al. 1996; Trivedi et al. 2006). Furthermore, interindividual differences can give rise to sometimes severe adverse drug reactions (ADRs) in a subset of patients that overall account for approximately 7% of all hospitalizations and 0.3% of death among all hospitalized patients (Lazarou et al. 1998; Pirmohamed et al. 2004). Among the factors causing interindividual differences, genetic germline variations in genes that are involved in pharmacokinetics and pharmacodynamics are estimated to explain 20–30% of drug response variability.

Notably, many of these pharmacogenes are among the most polymorphic genes in the human genome and harbor thousands of genetic variants, which can change enzyme activity or disrupt drug-target interactions, thereby eventually altering drug effects (Lauschke et al. 2017; Zhou et al. 2021a). Much effort has been made to identify actionable associations between genetic variants and differential drug response. As of 2021, > 310 drugs have received pharmacogenomic information in their labels or have received guidelines by pharmacogenomic expert working groups, such as the Clinical Pharmacogenetics Implementation Consortium (CPIC) and the Dutch Pharmacogenetics Working Group (DPWG), that can guide drug selection or posology (Lauschke et al. 2019; Shekhani et al. 2020).

Nevertheless, only a fraction of these established pharmacogenomic biomarkers is implemented in routine clinical care and the only preemptive tests that are mandated are for HLA-B*57:01 and *DPYD* variants to inform abacavir and fluoropyrimidine therapy, respectively. While the underlying reasons are complex and multifaceted, prevalence of the variants in question constitutes one of the factors that impacts the clinical utility of genetic testing (Lauschke and Ingelman-Sundberg 2016; Russell et al. 2021). Thus, mapping variant frequencies in different ethnogeographic groups can provide important information to inform cost-effectiveness modeling and guide population-specific genotyping strategies. Here, we provide an updated overview of population pharmacogenomics of ten important pharmacokinetic genes (*CYP2D6*, *CYP2C19*, *DPYD*, *TPMT*, *NUDT15* and *SLC22A1*), drug targets (*CFTR*) and genes involved in adverse event risk independent of drug pharmacokinetics or target (*HLA-A*, *HLA-B* and *G6PD*). We provide a detailed overview of ethnogeographic differences in allele frequencies, infer functional consequences and discuss implications and relevance for the implementation of population-specific precision public health. For other clinically relevant pharmacogenes, such as *CYP2B6* (Langmia et al. 2021), *UGT1A1* (Hall et al. 1999) or *NAT2* (Sabbagh et al. 2011), we refer the interested reader to excellent reviews on the topic.

## *CYP2D6*

CYP2D6 is one of the most pleiotropic drug-metabolizing enzymes and is involved in the hepatic clearance of approximately 25% of all clinically used drugs, including tricyclic antidepressants, opioids, antiemetics and antiarrhythmics (Zanger and Schwab 2013). Importantly, at least in part due to the lack of important endogenous substrates and low evolutionary constraints, *CYP2D6* constitutes one of the most polymorphic genes in the cytochrome P450 (*CYP*) gene family, resulting in drastic functional diversity of CYP2D6 (Fujikura et al. 2015; Ingelman-Sundberg 2005). Of the more than 100 different *CYP2D6* alleles that have been described to date, the loss-of-function (LOF) alleles CYP2D6*3, *4, *5 and *6, the decreased function alleles *9, *10, *17, *29 and *41 as well as the *CYP2D6* duplications *1xN and *2xN are functionally most relevant and are common with minor allele frequencies (MAF) > 1% in at least one population (Tables 1 and 2). Over the past decades, substantial interethnic differences have been revealed for these alleles, which translate into substantial variability in metabolic phenotypes across populations (Gaedigk et al. 2017; Zhou et al. 2017).

**Table 1:** Frequencies of common *CYP2D6* LOF alleles across countries/populations

Country	*n*	AF, %			
		*3	*4	*5	*6
Europe					
Austria	93	0.5	14	1.6	0.5
Croatia	1119–2637	2.3	16.7	1	1.1
Czech Republic	42–265	1.6	21.6	3.1	0.1
Denmark	225–634	2.2	20.5	5.9	1.4
Estonia	2394–44,448	1.8	16.7	1.5	0.9
Finland	12,589–13,956	3.5	10	2.2	2.1
Germany	195–1758	1.1	19.6	3.2	1
Greece	327	2.1	17.7	N/A	N/A
Hungary	55–591	1.6	19	1.8	0.9
Italy	110–917	1	16.4	2.4	0.9
Netherlands	105–1158	1.5	18.9	7.2	0.6
Norway	83–403	0	21.1	6	0.9
Poland	166–791	1.6	20.8	N/A	N/A
Portugal	100–1138	0.7	17	2.6	1
Russia	290–1663	1.2	17.6	1.6	1
Slovakia	26	2	28	N/A	N/A
Spain	51–2328	1.2	18.6	2.3	0.6
Sweden	205–2020	1.6	20.7	4.1	0.6
Turkey	100–785	0.7	13.2	1.8	1.1
UK	91–168	3.3	24.2	N/A	N/A
Americas					
Alaska	94	0	14.4	N/A	N/A
Brazil	33–1020	1.5	10.5	4	0.8
Canada	39–110	0	8.5	N/A	N/A
Columbia	121	1.2	19.4	0.8	0
Costa Rica	49–197	0.6	15.8	4.1	0.3
Cuba	126–130	0	14.5	1.8	0.6
Mexico	74–291	0.3	7.7	1.6	0.1
Nicaragua	98–137	1	15.1	4	0
Panama	105–136	0	15.4	0	0.8
US	104,509	1.4	16.1	3.4	1
US–African American	6762	0.3	4	5.3	0.3
US–Asian	251	0.4	5.2	2.8	0.2
US–Caucasian	37,571	1.6	18.7	3.1	1.2
US–Hispanic	2072	0.8	12.7	2.9	0.7
Venezuela	24–179	0	12.6	0.6	0.4
Africa					
Egypt	145		9.6	2	N/A
Ethiopia	69–122	0	3.4	3.3	0
Ghana	193	0	7	6	0
Kenya	195	N/A	3.3	2.8	N/A
Zimbabwe	114	0	2	4	N/A
East Asia					
China	100–1954	0	0.5	7.7	0
Japan	82–455	0	0.8	4.9	0
South Korea	49–1897	0	0.2	4.9	0
South Asia					
India	160	0.3	10.3	1.9	0
Indonesia	144	1.4	N/A	2.1	N/A
Thai	920	N/A	1.3	6.7	N/A
Vietnam	136	N/A	0.7	8.1	N/A
Middle East					
Iran	100	0.5	10.8	3	0.5
Qatar	107	N/A	10.3	N/A	N/A
Saudi Arabia	101–192	0	3.5	1	0
Syria	51	0	9.8	0.98	0.98
United Arab Emirates	151	N/A	9	N/A	N/A
Oceania					
Australia	5408	1.6	17.8	2.9	0.7
Australia aborigines	239	0	1.5	7.5	0
New Zealand	60	0.9	7.9	1.8	0
Papua New Guinea	84–88	0	1.5	5.4	0

The corresponding references are provided in Supplementary Table 1

*AF* allele frequency, *n* number of individuals genotyped, *N/A* not available

**Table 2:** Frequencies of decreased (*9, *10, *17, *29 and *41) and increased (duplications) function *CYP2D6* alleles across countries/populations

Country	*n*	AF, %					
		*9	*10	*17	*29	*41	Duplication (*1xN, *2xN)
Europe							
Austria	93	1.6	4.3	0	0	12.4	1.6
Croatia	1119–2637	N/A	N/A	N/A	N/A	10.8	3.4
Denmark	225–634	3.4	1.3	0	N/A	8.4	0.8
Estonia	2373–44,448	2	N/A	N/A	N/A	5.8	0.3
Finland	56–13,956	1.3	1.1	0	N/A	3.2	4.3
Germany	195–1758	1.9	1.7	0	N/A	8.4	1.3
Greece	327	N/A	N/A	N/A	N/A	N/A	6
Hungary	55–591	1.5	1.8	0	0	7.5	1.8
Italy	174–917	3	1.7	2.6	0.3	0.3	15.2
Netherlands	105	3	N/A	N/A	N/A	15	2.9
Portugal	1138	2.5	2.5	2.2	N/A	6.7	3
Russia	290–1663	N/A	4.1	N/A	N/A	7.6	2.4
Spain	51–2328	2.5	1.7	0.9	0	4.7	3.5
Sweden	1816–12,993	2.5	N/A	N/A	N/A	7.4	0.5
Turkey	100–785	0.6	7.7	1.1	N/A	N/A	5.6
Americas							
Alaska	94	0	4.3	N/A	N/A	4.3	N/A
Brazil	87–1020	1.3	2.2	4.8	2.8	6.2	1.8
Canada	39–110	N/A	3.6	N/A	N/A	N/A	N/A
Columbia	121	N/A	N/A	1.6	N/A	N/A	N/A
Costa Rica	49–197	N/A	0.9	4.1	2.2	3.4	3.8
Cuba	126–130	N/A	0.6	6.4	N/A	N/A	N/A
Mexico	74–243	0.4	2.9	0.5	0.1	1.2	2.8
Nicaragua	98–137	4.4	3.2	0	N/A	N/A	N/A
US	104,509	2.4	1.7	2.7	1.4	8.2	0.9
US–African American	6762	0.4	3.7	16.8	9.4	2.5	1
US–Asian	251	0.8	9.2	0.2	0	4.4	1
US–Caucasian	37,571	2.9	1.4	0.3	0.1	9.6	0.9
US–Hispanic	2072	1.7	1.4	1.9	1.6	5.7	0.9
Venezuela	24–179	N/A	3.2	N/A	N/A	N/A	N/A
Africa							
Egypt	145	N/A	3.4	N/A	N/A	15.1	N/A
Ethiopia	69–122	N/A	8.5	11.2	N/A	22.9	N/A
Ghana	193	0	3.1	27.7	N/A	N/A	N/A
Kenya	195	N/A	1	20.3	15.1	N/A	0.5
Zimbabwe	76–114	N/A	N/A	34	N/A	N/A	N/A
East Asia							
China	100–1954	0	43.4	0	0	3.3	0.43
Japan	86–455	0	37	0	0	1.3	0.8
South Korea	49–1899	0	46.9	0	0	1.8	0
South Asia							
India	160	0	5.9	0	0	12.5	2.2
Indonesia	144	N/A	28.8	N/A	N/A	4.5	N/A
Thai	920	N/A	49.6	N/A	0.1	6.5	N/A
Vietnam	136	N/A	43.8	N/A	N/A	N/A	N/A
Middle East							
Iran	100	N/A	9	0	N/A	N/A	N/A
Qatar	107	N/A	N/A	N/A	N/A	15	N/A
Saudi Arabia	101–192	N/A	3	3	2.9	18.4	10.4
Syria	51	0	2.9	0	N/A	N/A	7.8
United Arab Emirates	151	N/A	3.3	2.5	1.6	15.2	5.9
Oceania							
Australia	5408	2.3	3.3	0.2	N/A	10.2	1.8
Australia aborigines	239	0	0.8	0.2	N/A	N/A	N/A
New Zealand	60	0	6.1	N/A	N/A	3.5	N/A
Papua New Guinea	84–88	0	1.5	0	0	0	12

The corresponding references are provided in Supplementary Table 1

*AF* allele frequency, *n* number of individuals genotyped, *N/A* not available

The splice variant CYP2D6*4 (rs3892097) constitutes the globally most common *CYP2D6* variant allele of functional importance. In Europe, CYP2D6*4 is prevalent across North and Central Europe with frequencies around 20–25%. The highest frequency of this allele was observed on the Faroe Islands (33.4%), whereas it is substantially less prevalent in Southern Europe in Italy (16.4%), Greece (17.7%) and Turkey (13.2%), resulting in a European North-to-South gradient (Petrović et al. 2020). In addition, high CYP2D6*4 frequencies were observed in Ashkenazi Jews (22.6%), a genetic isolate historically living in Europe that harbors a genetic repertoire that is distinctly different from other populations (Scott et al. 2007; Zhou et al. 2018). CYP2D6*4 is also abundant in American populations, particularly in Columbia (19.4%) (Isaza et al. 2000), Costa Rica (15.8%) (Céspedes-Garro et al. 2014), Panama (15.4%) (Jorge et al. 1999) and Nicaragua (15.1%) (Agúndez et al. 1997). CYP2D6*4 frequencies are slightly lower in West Asia (7.8%) and Central-South Asia (8.5%), whereas the variant is almost absent among East Asians (0.6%) (Gaedigk et al. 2017). Similarly, CYP2D6*4 frequencies are lower in Africa, ranging from 2% in Zimbabwe (Dandara et al. 2001) to 7% in Ghana (Griese et al. 1999). Interestingly, CYP2D6*4 frequency in African Americans (6.3%) is higher than in most African populations that reside in Africa (3.3%), possibly at least in part because of genetic admixture.

Besides CYP2D6*4, also other *CYP2D6* LOF alleles such as CYP2D6*3 (rs35742686) and *6 (rs5030655) are most abundant in European populations. The frequencies of CYP2D6*3 are above 1% in most European countries with the highest values found in Finland (3.5%) and the United Kingdom (3.3%) (Auton et al. 2015). In contrast, the allele is rare or absent in Portugal (0.7%) (Albuquerque et al. 2013; Correia et al. 2009), Turkey (0.7%) (Aydin et al. 2005; Aynacioglu et al. 1999; Mizzi et al. 2016; Serin et al. 2012), Austria (0.5%) (Beer et al. 2011) and Norway (0%) (Molden et al. 2002). Notably, while CYP2D6*3 is overall less abundant outside Europe, it is also found in countries with admixed populations, such as Brazil (Friedrich et al. 2014; Kohlrausch et al. 2009). Similarly, CYP2D6*6 is only common in some European populations with frequencies up to 2.1% in Finland.

In contrast to the LOF alleles CYP2D6*3, *4 and *6, deletion of *CYP2D6* (CYP2D6*5) is most common in Africa, East Asia and Oceania with frequencies pivoting around 5–6% (Gaedigk et al. 2017). In Europe, CYP2D6*5 prevalence is overall lower with a South-East to North-West gradient, ranging from 1% in Croatia (Ganoci et al. 2017) to 7.2% in Netherlands (Poulussen et al. 2019). Frequencies of CYP2D6*5 are similarly low in the Americas (2.1%), as well as in South Asian populations (3.2%) with national frequencies up to 5.1% in Malaysia (Teh et al. 2001).

The reduced function variant CYP2D6*10 (rs1065852, rs1135840) is the most common allele in East Asians with frequencies up to 64.1% (Qin et al. 2008). Frequencies are high in Han Chinese (43.5%) and Hui (51%), but substantially lower in Mongolians (25.2%) and Tibetans (28.1%) (Yin et al. 2012). In contrast, CYP2D6*10 is substantially less prevalent in African (6.6%), Ashkenazim (6.2%), European (2.8%) and American populations (2.6%) (Gaedigk et al. 2017). The inframe deletion variant CYP2D6*9 (rs5030656) is globally rare but relatively common in European and American populations with highest frequencies in Denmark (3.4%) (Pedersen et al. 2005; Rasmussen et al. 2006) and Nicaragua (4.4%) (Agúndez et al. 1997).

CYP2D6*17 (rs16947, rs28371706) and CYP2D6*29 (rs16947, rs1135840, rs61736512, rs59421388) are both African-specific alleles with frequencies of 9–34% (Aklillu et al. 1996; Dandara et al. 2001; Masimirembwa et al. 1996) and 4–20% (Dodgen et al. 2016; Wennerholm et al. 2001), respectively. Although considered extremely rare in other populations, they have been also identified in admixed populations. In the Americas, both alleles were prevalent in the Afro–Trinidadian population (CYP2D6*17, 16.5%; CYP2D6*29, 8.7%) (Montané Jaime et al. 2013), as well as in Cuba (CYP2D6*17, 6.4%) (Llerena et al. 2012), Brazil (CYP2D6*17, 4.8%; CYP2D6*29, 2.8%) (Antunes et al. 2012; Friedrich et al. 2014; Kohlrausch et al. 2009) and Costa Rica (CYP2D6*17, 4.1%; CYP2D6*29, 2.2%) (Céspedes-Garro et al. 2014). In addition, both CYP2D6*17 and CYP2D6*29 were observed in the Middle East with frequencies pivoting around 2.5% and 1.6%, respectively (Khalaj et al. 2019; Luo et al. 2004; McLellan et al. 1997; Qumsieh et al. 2011; Sistonen et al. 2007).

The splicing variant CYP2D6*41 (rs28371725) is globally common with highest frequencies being reported in Bedouins (29%) (Luo et al. 2004) and Indians (12.5%) (Sistonen et al. 2009). CYP2D6*41 is also prevalent in African (9.7%), European (7.4%), American (3.7%) and East Asian (2.2%) populations albeit at overall lower frequencies. Notably, however, CYP2D6*41 frequencies can be substantially higher than the respective superpopulation averages, as observed in Ethiopia (22.9%) (Aklillu et al. 2002), Italy (15.2%) (Carano et al. 2018) and the Netherlands (15%) (Poulussen et al. 2019).

In contrast to the aforementioned decreased function and LOF *CYP2D6* alleles, the gain-of-function (GOF) duplication allele CYP2D6*1xN is most prevalent in Oceanian Aborigines (11.5%) (Sistonen et al. 2007), particularly in Papua New Guinea (12%) (von Ahsen et al. 2010), whereas the genetically distinct GOF allele CYP2D6*2xN is most common in the Mozabite population in North Africa (28.3%) (Sistonen et al. 2007). In contrast, in Sub-Saharan Africa, the frequencies of *CYP2D6* duplications are overall low (2.4% and 0.8% for CYP2D6*1xN and CYP2D6*2xN, respectively) (Sistonen et al. 2007). *CYP2D6* duplications are moreover common in Ashkenazim and Middle Eastern populations with combined frequencies of 8% and 3.9% (Fuselli et al. 2004; Scott et al. 2007). *CYP2D6* gene duplications are rare in Central European populations, such as Germans (1.3%), Austrians (1.6%) and Hungarians (1.8%), but considerably higher in both Northern and Southern European groups, such as Finnish (4.3%), Spanish (3.5%), Greek (6%) and Turkish (5.6%) (Petrović et al. 2020). In Asian populations, both CYP2D6*1xN and CYP2D6*2xN are rare with frequencies below 1% (Sistonen et al. 2007).

**Fig. 1:**
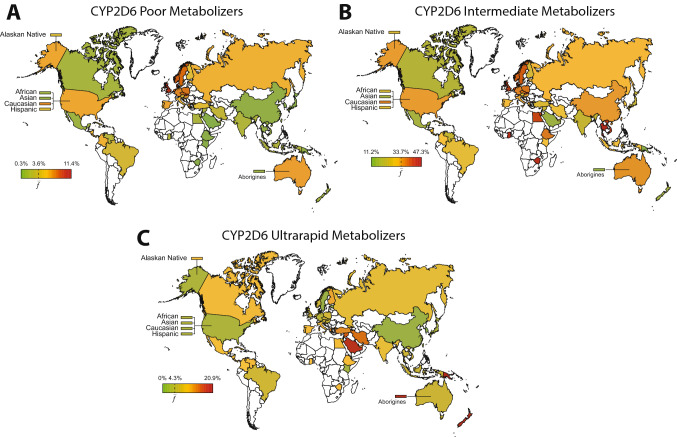
Global distribution of inferred CYP2D6 phenotypes. Frequencies of CYP2D6 poor metabolizer (**A**), intermediate metabolizer (**B**) and ultrarapid metabolizer (**C**) phenotypes were calculated based on the frequencies of loss-of-function alleles (*3, *4, *5 and *6), decreased function alleles (*9, *10, *17, *29 and *41) and increased function alleles (*1xN and *2xN) from 53 countries/populations (Tables 1 and 2; Supplementary Table 1). Countries are color-coded with the highest frequency in red, the average frequency across all populations ($\overline{f }$) in yellow, and the lowest frequency in green. In case of missing population frequencies, averaged continent frequency data from the literature (Gaedigk et al. 2017) were used to infer metabolizer phenotypes

The country-specific *CYP2D6* allele frequency data can be aggregated to infer CYP2D6 phenotypes (Gaedigk et al. 2017; Koopmans et al. 2021). The frequency of CYP2D6 poor metabolizers (PM), defined as individuals carrying two LOF alleles, is highest in Ashkenazi Jews (6%) and European population (5.4–11.4%) and lowest in populations from the Middle East (0.9%), East Asia (0.4%) and Oceania (0.4%; Fig. 1). In contrast, the prevalence of intermediate metabolizers (IM) that exhibit reduced but measurable CYP2D6 metabolism was found to be highest in African populations (10–60%) and Ashkenazim (10–40%), and lowest in South Asia (3.8%) and the Americas (2.8%). Ultrarapid metabolizers (UM) that carry at least one functional gene duplication, are most common in indigenous Oceanian populations (21.2%) and North Africa (up to 39%), whereas they are lowest in East Asia (1.4%). These functional extrapolations can provide important information for population-specific drug selection and the posology of CYP2D6 substrates.

## *CYP2C19*

CYP2C19 is a key enzyme involved in the metabolism of the antiplatelet drug clopidogrel, selective serotonin reuptake inhibitors (SSRIs) as well as proton pump inhibitors, and genetic variability in *CYP2C19* contributes to the differential response to these substrates. The clinically most relevant variant alleles are CYP2C19*2 (rs4244285) and CYP2C19*3 (rs4986893) that abolish enzyme activity and the regulatory CYP2C19*17 variant (rs12248560) that results in increased gene activity (Table 3).

**Table 3:** Frequencies of common functional *CYP2C19* alleles across countries/populations

Country	*n*	AF, %		
		*2	*3	*17
Europe				
Croatia	1119–1247	15.2	N/A	23.5
Czech Republic	42–265	8	N/A	29
Denmark	69–634	15.8	0	20.1
Estonia	2407–44,448	13.5	0	26.4
Finland	177–13,956	17.5	0	19.6
Germany	60–1758	14.9	1.8	24.9
Greece	283–327	14.1	0	18.2
Hungary	112–591	13.3	0.9	23
Italy	360–917	11.8	0	22.1
Netherlands	428–1158	14.1	0.2	19
Norway	83–403	15.3	0.3	22
Poland	166–791	16.3	N/A	29.8
Portugal	33–400	13.4	0	28.8
Russia	290–1663	13.6	0.3	15
Slovakia	26	19	N/A	33
Spain	78–2328	14	0	17.1
Sweden	110–2020	14	0.5	19.2
Turkey	96–785	11.3	1.8	24
UK	91–168	13.4	N/A	24.2
Americas				
Brazil	1034–1212	14.8	0	19.2
Columbia	189–239	8.7	0	17.6
Costa Rica	141	7.1	0	10.3
Cuba	267	10.1	2.6	16.1
Mexico	238	8.6	0	14.3
Nicaragua	212	8.3	N/A	6.8
US	3342	16.5	0.1	20.3
US–African American	250	19.4	0.4	18.2
US–Asian	250	27.6	4.8	6.2
US–Caucasian	250	13.2	0.4	15.8
US–Hispanic	250	12.8	0	15.2
US–Native	50	11	0	9
Venezuela	680	7.3	2.3	N/A
Africa				
Egypt	247	11	0.2	N/A
Ethiopia	70–190	13.3	2.3	13.2
Ghana	169	5.9	0	N/A
Morocco	290	11.4	0	N/A
Tanzania	251	17.9	0.6	N/A
Uganda	99	12.6	1	17.2
Zimbabwe	168	4.2	N/A	N/A
East Asia				
China	824–5679	29.1	4.4	1.2
Japan	84–1194	30	11.3	1.1
South Korea	271–648	28.3	8.6	1.5
South Asia				
India	206–600	35.2	2.4	19.2
Indonesia	96–166	34	6.9	4.7
Thai	1051	27	6	4
Vietnam	100	20.5	2.5	1
Middle East				
Iran	82–200	12.9	0.7	21.6
Qatar	108–976	11.6	0.3	20.4
Saudi Arabia	97–201	11.2	0	25.7
Oceania				
Australia	5408	16.4	0.2	20.2
Australia aborigines	239	35.5	14.3	N/A
New Zealand	312	18.9	N/A	> 17
Papua New Guinea	172–401	45	16.9	N/A

The corresponding references are provided in Supplementary Table 2

*AF* allele frequency, *n* number of individuals genotyped, *N/A* not available

CYP2C19*2 is globally common with highest frequencies found in Oceanian (61%) (Scott et al. 2013) and Asian populations (28.4% in East Asian and 31.8% in South Asian)(Ionova et al. 2020). On a per-country level, CYP2C19*2 was most prevalent in the Vanuatu atoll with a reported frequency of 71% (Kaneko et al. 1997, 1999). In Africa, the Americas and Europe, the frequencies of this allele pivot around 12–15% (Scott et al. 2013) with South African Xhosa (21%), Cypriots (21%), Romani (20.8%) and Maltese (20%) constituting the ethnogeographic hotspots (Drögemöller et al. 2010; Pimenoff et al. 2012; Sipeky et al. 2013; Mizzi et al. 2016).

Like CYP2C19*2, also the CYP2C19*3 allele is common in Oceania (15%) (Scott et al. 2013) and across East Asia (6%) (Ionova et al. 2020). Notably, the frequency of CYP2C19*3 in East Asia exhibits an East-to-West gradient with highest frequencies in Japanese (11.3%), followed by South Koreans (8.6%) and Chinese (4.4%) (Dorji et al. 2019). Interestingly, while CYP2C19*2 and CYP2C19*3 are both common across Oceania, their frequencies in Polynesian populations, including Samoan, Tongan, Fijian, Cook Islander and Maori, are substantially lower than in Melanesians, including Papua New Guinean, Vanuatuan and Aboriginal Australian (CYP2C19*2: 22% in Polynesians vs. 51% in Melanesians; CYP2C19*3: 4% in Polynesians vs. 19% in Melanesians) (Helsby 2016).

CYP2C19*17 is prevalent worldwide with frequencies above 15% except for East Asian populations (3.7%) (Ionova et al. 2020). In Europe, the highest prevalence was reported in Slovakia (33%), Poland (29.8%) and the Czech Republic (29%), whereas frequencies are lower in South and East Europe (Cyprus, 11%; Span, 17%; Russia, 15%) (Gawrońska-Szklarz et al. 2012; Mizzi et al. 2016; Vicente et al. 2014).

**Fig. 2:**
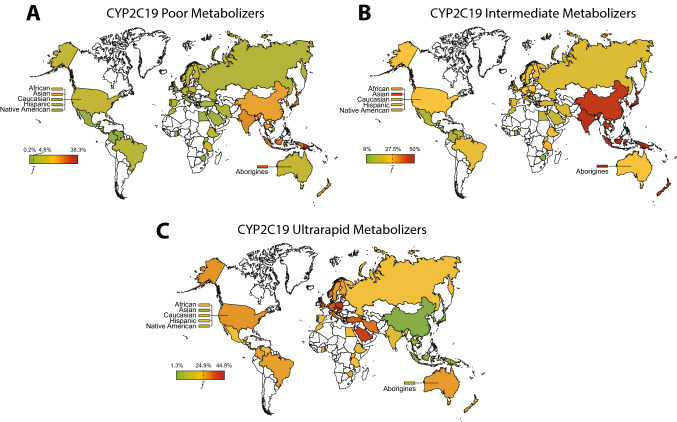
Global distribution of inferred CYP2C19 phenotypes. Frequencies of CYP2C19 poor metabolizers (**A**), intermediate metabolizers (**B**) and ultrarapid metabolizers (**C**) were calculated based on frequencies of the loss-of-function alleles CYP2C19*2 and *3, as well as the increased function allele CYP2C19*17 for 52 countries/populations (Table 3; Supplementary Table 2). Countries are color-coded with the highest frequency in red, the average frequency across all populations ($\overline{f }$) in yellow, and the lowest frequency in green. In case of missing population frequencies, averaged continent frequency data from the literature (Ionova et al. 2020; Scott et al. 2013) were used to infer metabolizer phenotypes

The functional allele frequency data has been used to predict CYP2C19 phenotypes across ethnicities (Koopmans et al. 2021). CYP2C19 PM status was most common in Oceania where around 58% of individuals are homozygous or compound heterozygous for *CYP2C19* LOF alleles (Fig. 2). Considerable numbers of CYP2C19 PMs were also reported in East Asian (14.2%) and Central/South Asian (11.8%) populations, whereas their numbers are lower in Latin America (1.1%), Europe (2.7%) and Africa (3.3%). CYP2C19 UMs are most common in European, African and Latin American populations with frequencies pivoting around 20–30%, whereas only 2.1% of the East Asians are UMs (Koopmans et al. 2021).

## *DPYD*

Fluoropyrimidines, including 5-fluorouracil and its prodrugs capecitabine and tegafur, are important chemotherapeutics for the treatment of various solid tumors. They are among the most prescribed anticancer drugs worldwide with more than two million patients estimated to use fluoropyrimidines each year (Ezzeldin and Diasio 2004). However, up to 40% of patients experience fluoropyrimidine-induced toxicity that is severe enough to require discontinuation of therapy, and in 0.5–1% of patients these ADRs are fatal (Hoff et al. 2001; Van Cutsem et al. 2001). The toxicity of fluoropyrimidines is strongly associated with the metabolic activity of dihydropyrimidine dehydrogenase (DPD), the enzyme catalyzing the rate-limiting step in the biotransformation of fluoropyrimidines into non-toxic metabolites. As such, reduced activity of DPD increases fluoropyrimidine exposure, resulting in increased cytotoxicity.

Interindividual variation in DPD activity is strongly associated with genetic variability of the respective gene, *DPYD*. The most well-studied *DPYD* variant is DPYD*2A (rs3918290; c.1059 + 1G > A; IVS14 + 1G > A), a splicing variant that results in exon skipping and gives rise to a truncated gene product with no catalytic activity (Vreken et al. 1996). The highest frequency of DPYD*2A is found in the Finnish population (2.4%) (Zhou et al. 2020), whereas frequencies in Central, South and East Europe are > twofold lower, pivoting around 1%, 0.5% and 0.3%, respectively (Raida et al. 2001; Salgueiro et al. 2004; Sulzyc-Bielicka et al. 2008; Uzunkoy et al. 2007; van Kuilenburg et al. 2001) (Table 4). DPYD*2A is extremely rare in Asian, African and indigenous American populations (Elraiyah et al. 2017; Hariprakash et al. 2018; Zhou et al. 2020).

**Table 4:** Frequencies of DPYD*2A and *HapB3* alleles across selected countries

Country	*n*	*2A (AF, %)	*n*	*HapB3* (AF, %)
Europe				
Bulgaria	1334	0.4	N/A	
Czech Republic	422	0.4	N/A	
Estonia	2414	0.9	2289	2
Finland	12,560	2.4	1737	1.2
France	3680	0.3	3680	2
Germany	851	0.9	453	3.3
Netherlands	1357	0.9	191	2.6
Poland	252	0.2	N/A	
Portugal	73	0.7	N/A	
Sweden	13,064	0.8	1000	1.9
Turkey	218	0.6	N/A	
Asia				
China	117	0	N/A	
India	2000	0.05	2000	1.4
Japan	107	0	N/A	
South Korea	1907	0	N/A	
Africa				
Egypt	239	0	N/A	
Americas				
Argentina	102	0.5	N/A	
Brazil	1171	0.1	1171	0.4
Canada	2617	0.5	N/A	

The corresponding references are provided in Supplementary Table 3

*AF* allele frequency, *n* number of individuals genotyped, *N/A* not available

In addition to DPYD*2A, the *DPYD* haplotype HapB3, comprising three intronic variants (c.483 + 18 G > A/rs56276561, c.680 + 139 G > A/rs6668296 and c.959-51T > C/rs115349832) and one synonymous variant (E412E; c.1236 G > A; rs56038477), has been associated with severe fluoropyrimidine toxicity (Amstutz et al. 2009). This association is likely due to c.1129–5923C > G (rs75017182), a deep intronic variant that is in strong linkage with HapB3 and that impairs DPD function by affecting pre-mRNA splicing (van Kuilenburg et al. 2010). Importantly, c.1129–5923C > G/HapB3 is common in many populations. In Europe, it is considered the most common reduced function *DPYD* variant with an averaged frequency of 2.1% and highest prevalence in the Netherlands (2.6%) and Germany (3.3%) (van Kuilenburg et al. 2010; Zhou et al. 2020). In contrast, HapB3 is less frequent in Africa (0.2%), East Asia (0.2%), Latinos (0.8%) and Ashkenazim (0.7%) (Zhou et al. 2020). Another well-established decreased function *DPYD* variant is p.Y186C (rs115232898), a variant that is prevalent with frequencies up to 3.3% among individuals of African ancestry but is almost absent in other populations (Offer et al. 2013). Other functionally relevant *DPYD* variants, such as p.D949V (rs67376798), are rare with frequencies below 1% in all populations.

Previous estimates for the global prevalence of partial and full DPD deficiency are 3–8% and 0.02–0.2%, respectively, with highest frequencies in Africans and Finnish and lowest in Ashkenazi Jews and East Asians (Caudle et al. 2013; Zhou et al. 2020). As frequencies of DPD deficiency differ by up to tenfold between populations, these data thus emphasize the importance of population-adjusted strategies for the optimization of fluoropyrimidine dosing and solid cancer therapy.

## *TPMT* and *NUDT15*

Thiopurine methyltransferase (encoded by *TPMT*) and nudix hydrolase 15 (encoded by *NUDT15*) play important roles in the metabolism of the thiopurines mercaptopurine and thioguanine, which are widely used in the treatment of acute lymphoblastic leukemia, inflammatory bowel diseases and autoimmune disorders. Thiopurines are metabolized intracellularly into thioguanosine monophosphate (TGMP), which is further converted into the active thioguanine di- and triphosphates that exert their cytotoxic and antiproliferative effects by blocking purine synthesis and by causing direct damage to DNA and RNA (Bökkerink et al. 1993; Inamochi et al. 1999; Karim et al. 2013). Furthermore, they have anti-inflammatory effects by inducing T cell apoptosis via inhibition of the GTPase RAC1 (Poppe et al. 2006). TPMT plays a central role in the metabolism of thiopurines into inactive methyl-metabolites thereby shunting TGMP away from further metabolic activation. Similarly, NUDT15 dephosphorylates thioguanine di- and triphosphates back into its monophosphate form, counteracting its incorporation into DNA and RNA.

Genetic variations can cause TMPT and NUDT15 deficiency, resulting in excessive formation of thioguanine di- and triphosphates and an increased risk of severe myelosuppression. The most common and well-characterized *TPMT* alleles are TPMT*3A (rs1142345 and rs1800460), *3C (rs1142345) and *2 (rs1800462), which together explain more than 90% of decreased TPMT activity phenotypes (Schaeffeler et al. 2004; Zhou et al. 2020). TPMT*3A is most common in European and Latin American populations with frequencies pivoting around 2–4%. The highest TPMT*3A frequencies in Europe were observed in the UK (4.5%) (Ameyaw et al. 1999) and Greenland (8.1%) (Toft et al. 2006), whereas frequencies in Croatia were substantially lower (1.9%) (Ladić et al. 2016). No TPMT*3A alleles were found in 194 indigenous Saami in Norway (Loennechen et al. 2001). In Latin America, frequencies were highest in Brazil (up to 3.9%) (Ferreira et al. 2020), Colombia (3.6%) (Isaza et al. 2003) and Argentina (3.1%) (Laróvere et al. 2003).

In Asian and African populations TPMT*3A is very rare and instead TPMT*3C is the predominant allele underlying TPMT deficiency (Chang et al. 2002; Hon et al. 1999). In Asia, frequencies of TPMT*3C range between 0.8% in Japanese, 0.9% in Koreans (Lee et al. 2008), 1.3–3% in Chinese populations and 0.8–2.8% across South Asia (Hiratsuka et al. 2000; Kham et al. 2002; Lee et al. 2008; Zhang et al. 2003). These allele-specific interethnic differences are even more striking in Sub-Saharan Africa where TPMT*3C is highly abundant in Ghana (7.6%) (Ameyaw et al. 1999), Kenya (5.4%) (McLeod et al. 1999) and Nigeria (5.3%) (Adehin et al. 2017), but relatively rare in North African populations, such as Tunisians (1.4%) (Melaouhia et al. 2012), Egyptians (1.3%) (Hamdy et al. 2003) and Libyans (1%) (Zeglam et al. 2015). The other reduced function variant, TPMT*2, is globally rare with MAF < 1% with few reported exceptions, such as in Iran (2.2%)(Bahari et al. 2010) and Sardinia (1.7%) (Rossino et al. 2006).

Based on frequencies of TPMT*3A, *3C, *2, it is estimated that the frequency of patients harboring intermediate TPMT activity is around 3–14%, and approximately 1 in 178 to 1 in 3,736 patients are fully TPMT deficient (Relling et al. 2011). When extending these analyses using Next Generation Sequencing to also include other functional variations, recent studies suggested highest prevalence of intermediate and full TMPT deficiency in Africa with frequencies of 11% and 0.3%, respectively, whereas the corresponding frequencies were lowest in Asian populations (0.03–0.04% full deficiency; 3.3–3.9% intermediate activity) and Ashkenazim (0.02% full deficiency; 2.9% intermediate activity) (Zhou et al. 2020).

While polymorphisms in *TPMT* alone explain around 40% of thiopurine-induced ADRs (Schaeffeler et al. 2019), predictions can be further improved by including the missense variant p.R139C in *NUDT15* (c.415C > T; rs116855232) (Yang et al. 2015b, 2014). Mechanistically, this variant destabilizes the protein structure, thereby resulting in lower enzymatic activity (Rehling et al. 2021). p.R139C defines NUDT15*3 and is moreover part of NUDT15*2 in combination with the inframe deletion variant (rs746071566), in both cases resulting in a loss of gene product function. The frequency of p.R139C differs > 20-fold across populations. It is most abundant in Asian populations, including Japanese (16%) (Tanaka et al. 2015), Koreans (11.3%) (Kim et al. 2017), Chinese (12.7%) (Chao et al. 2017) and Indians (10.7%) (Shah et al. 2018), as well as Amerindian groups (5–32%) (Suarez-Kurtz et al. 2019). In contrast, frequencies are considerably lower in admixed Brazilian populations (6.8%) (Rodrigues et al. 2020) and across Europe (0.4%) with the exception of Nordic populations, such as Finns (2.3%) and Swedes (2%) (Wahlund et al. 2020). Similarly, p.R139C is almost absent in Africa and the Middle East (Jarrar and Ghishan 2019).

Due to the high frequency of p.R139C, NUDT15 deficiency is common in East Asian (22.6%), South Asian (13.6%) and Latin American (12.5–21.2%) populations (Moriyama et al. 2016), surpassing the prevalence of TPMT deficiency and suggesting that variations in *NUDT15* rather than in *TPMT* are the major drivers of thiopurine-induced toxicity across Asia and Latin America. In contrast, *TMPT* reduced function alleles explain the majority of thiopurine toxicity in Europe and Africa.

## *SLC22A1* (OCT1)

The *SLC22A1* gene encodes the organic cation transporter OCT1 that is highly expressed in hepatocytes, immune cells and most epithelial barriers. OCT1 partakes in the disposition of a large number of structurally diverse drugs (including metformin, tramadol, lamivudine, oxaliplatin, sorafenib and morphine), endogenous substrates (choline, acetylcholine and agmatine), vitamins (vitamin B1) and toxins (1-methyl-4-phenylpyridinium), and genetic variants in *SLC22A1* have been reproducibly associated with altered substrate pharmacokinetics (Arimany-Nardi et al. 2015; Chen et al. 2014; Herraez et al. 2013; Tzvetkov et al. 2013, 2011). Importantly, *SLC22A1* is highly polymorphic with more than 1,000 genetic variants of which 450 alter the amino acid sequence of the transporter (Schaller and Lauschke 2019). While most of these variations are very rare and poorly characterized, at least 15 functionally relevant alleles have been identified that are common in at least one population (Seitz et al. 2015).

In European populations, the reduced function alleles SLC22A1*2 (p.M420del; rs202220802) and SLC22A1*3 (p.R61C; rs12208357) constitute the most abundant alleles with frequencies of 10–20% and 2–10%, respectively (Schaller and Lauschke 2019; Zazuli et al. 2020). In addition, the LOF alleles SLC22A1*4 (p.G401S; rs34130495), SLC22A1*5 (p.G465R; rs34059508) and SLC22A1*6 (p.C88R; rs55918055) occur in Europe with frequencies of 1–7%, 0–8% and 0–2%. Notably, SLC22A1*4 seems to be graded from 7.1% in Spain, 5.4% in Sardinia and 4.2% among French Basques in the South of Europe to 1.6% in Finland, 2% in Britain and 0% on the Orkney islands in Northern Europe (Seitz et al. 2015). SLC22A1*7 to *15 are not found across Europe. In aggregate, these data indicate that around 44% of individuals of European descent carry at least one *SLC22A1* reduced function allele.

The patterns of genetic *SLC22A1* variability are substantially different in African populations. In Sub-Saharan Africa, SLC22A1*8 (p.R488M; rs35270274), a variant allele with slightly increased activity towards morphine and metformin, constitutes the most common allele with frequencies between 2 and 18% (Seitz et al. 2015). Furthermore, SLC22A1*7 (p.S14F; rs34447885) is common with frequencies up to 9%. Effects of this allele are substrate-specific, entailing reduced transport of metformin, tropisetron and tyramine, whereas no differences are observed for morphine, debrisoquine and tramadol. SLC22A1*2 is found across Sub-Saharan Africa albeit with lower prevalence than in Europe (0–11% compared to 10–20%). In aggregate, only around 15% of individuals in Africa harbor reduced function variants, whereas around 12% carry the African increased activity allele SLC22A1*8. In contrast to Sub-Saharan Africa, Northern Africa and the Middle East recapitulates the variant pattern observed in European populations with SLC22A1*2 and SLC22A1*3 being most common, while SLC22A1*7 and SLC22A1*8 are only rare with frequencies around 1%.

Compared to European and African populations, the genetic complexity in East Asian and indigenous American populations is considerably lower. In Pima, Maya, Surui and Colombian populations, OCT1 deficiencies are highly common with frequencies up to 94%, which is almost exclusively allotted to SLC22A1*2. In contrast, in East Asia, 95–98% of alleles are normactive with only few ethnogeographic hotspots of Asian-specific reduced activity variants, such as SLC22A1*12 (p.S29L; rs375175439) in She (10%), as well as SLC22A1*9 (p.P117L; rs200684404), SLC22A1*11 (p.I449T; rs183240019) and SLC22A1*15 (p.E284K) with frequencies of 5–6% in Mongolians, Nashi and Monghour in China, respectively (Chen et al. 2010; Cheong et al. 2011).

## Pharmacogenetically important *HLA* alleles

While around 80% of ADRs are consequences of excessive pharmacological actions, the remaining 20% are idiosyncratic events that are unrelated to the therapeutic effect of the drug (Uetrecht and Naisbitt 2013). Many but likely not all idiosyncratic ADRs are immunologically mediated and can affect virtually any tissue, either in isolation or in combination with systemic effects (Phillips 2016). Idiosyncratic ADRs are more often severe or life-threatening with specific manifestations, such as Stevens–Johnson syndrome (SJS) and toxic epidermal necrolysis (TEN) resulting in mortality rates up to 13–60% (Schulz et al. 2000; Watanabe et al. 2021). The human leukocyte antigen (*HLA*) gene family encodes the major histocompatibility complex (MHC), which regulates T-cell mediated immunity. *HLA* genes have been strongly implicated in the etiology of immune-related adverse events caused by a multitude of drugs (Lauschke et al. 2019). The established models suggest that drugs (1) act as haptens, binding covalently to proteins and forming new antigens, (2) directly interact with the T cell receptor via non-covalent bonds or (3) bind non-covalently to the MHC, resulting in deformations of the peptide-binding groove and altered antigen presentation (Pavlos et al. 2015).

Notably, *HLA* genes are extremely polymorphic, but most idiosyncratic immunological ADRs are restricted to carriers of one or few specific *HLA* variant alleles. For instance, abacavir binds exclusively to the peptide-binding groove of HLA-B*5701, resulting in altered presentation of self-peptides, which in turn triggers polyclonal alloreactive autoimmunity and gives rise to abacavir hypersensitivity syndrome (AHS)(Illing et al. 2012; Ostrov et al. 2012). Further prominent and clinically well-established associations are associations of allopurinol-induced cutaneous adverse events with HLA-B*58:01 and links between carbamazepine-induced SJS/TEN and HLA-B*15:02 and HLA-A*31:01.

**Fig. 3:**
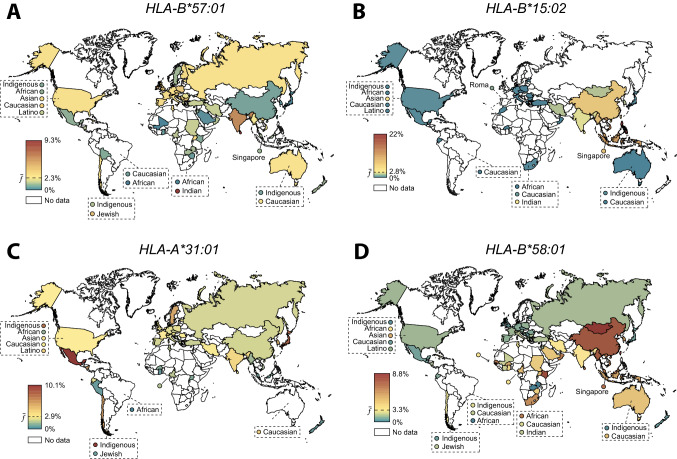
Global distribution of clinically important human leukocyte antigen (*HLA*) alleles. Allele frequencies of HLA-B*57:01 (**A**), HLA-B*15:02 (**B**), HLA-A*31:01 (**C**), and HLA-B*58:01 (**D**) across up to 74 countries are shown. Countries are color-coded with the highest frequency in red, the average frequency across all populations ($\overline{f}$) in yellow, and the lowest frequency in blue. Countries for which no *HLA* frequency information was available are colored white. Figure modified with permission from (Zhou et al. 2021b)

Abacavir is a nucleoside analog reverse transcriptase inhibitor that is used in combination with other antiretrovirals for the treatment of HIV/AIDS. In historic studies before the identification of HLA-B*5701 as a genetic risk factor, AHS occurs in around 5% of patients treated with abacavir with a mortality rate of around 3 per 1000 (Bannister et al. 2008; Hetherington et al. 2001). Importantly, while almost half of all HLA-B*57:01 carriers develop AHS after abacavir exposure, AHS was not observed in any of the patients without HLA-B*57:01 (Mallal et al. 2008). Based on these unambiguous data, preemptive testing of HLA-B*57:01 has become mandatory across the US and Europe before the initiation of abacavir therapy. HLA-B*57:01 allele frequency is a key factor to assess AHS risk in a population-scale. We recently evaluated the ethnogeographic distribution of pharmacogenetically relevant *HLA* alleles based on genetic information from 6.5 million individuals across 74 countries (Zhou et al. 2021b). The results showed that HLA-B*57:01 is generally rare in Africa, the Middle East and East Asia, whereas in Europe frequencies are reported between 1% in Sweden to 5.8% in Ireland (Fig. 3A). Globally, HLA-B*57:01 is most frequent in India (6.2%) and Sri Lanka (9.3%), whereas it is much less abundant in other South Asian countries such as Malaysia (1.1%), Thailand (2.1%) and Vietnam (2.6%).

Carbamazepine-induced severe cutaneous adverse reactions (SCAR) are associated with two alleles, HLA-B*15:02 and HLA-A*31:01, and odds ratios up to 2,504 (Chung et al. 2004) and 58 (Genin et al. 2014) have been reported, respectively. HLA-B*15:02 is exclusively found in Southeast Asian populations where allele frequencies are particularly high in the Philippines (22%), Vietnam (13.8%), Indonesia (11.6%) and Malaysia (11.5%), with the notable exception of Japan (< 0.1%; Fig. 3B). Consequently, genetic testing of HLA-B*15:02 is recommended in individuals of Asian ancestry but not for other populations. In contrast to the region-specific HLA-B*15:02, HLA-A*31:01 is common worldwide (Fig. 3C). It is most prevalent in indigenous populations in the Americas, such as in Argentina (28.8%), Mexico (10.1%), the United States (7.8%), Nicaragua (6.7%) and Chile (6.6%), whereas frequencies in Africa and Oceania seem to be lower (approximately 1%). However, frequency estimates of the latter are only based on small cohorts and further information in these populations is needed to corroborate these observations.

The xanthine oxidase inhibitor allopurinol is used for the treatment for hyperuricemia, but its utility is limited by the development of SCAR in up to 0.5% of patients (Yang et al. 2015a). HLA-B*58:01 is the predominant risk allele in Asian populations (Hung et al. 2005; Lonjou et al. 2008) where it is very common in Mongolia (8.8%), China (7.8%), Thailand (7.8%) and Singapore (7.2%; Fig. 3D). In addition, it is prevalent in several African countries, including Kenya (8.2%), Guinea Bissau (7.8%) and Senegal (6.9%). In contrast, HLA-B*58:01 frequencies are overall low across Europe and the Americas with frequencies ranging from 0.5 to 3.5%. Combined, these data provide the molecular basis for ethnogeographic differences in idiosyncratic ADR risk and suggest that preemptive testing can reduce idiosyncratic toxicity particularly in at-risk populations where the frequency of the respective *HLA* alleles are high.

## *CFTR*

The *CFTR* gene encodes a chloride channel that is part of the ATP-binding cassette (ABC) transporter superfamily (*ABCC7*). The gene product plays essential roles in ion and water secretion and absorption in epithelial tissues. Genetic variations that impact CFTR function constitute the cause of cystic fibrosis (CF), an autosomal recessive disorder most commonly observed in populations of European descent. CF manifests primarily as lung disease with symptoms that resemble pneumonia, bronchiectasis and asthma. Further non-pulmonary symptoms include pancreatic dysfunction, intestinal obstructions and elevated sweat electrolytes. Notably however, phenotypes, ages of onset and clinical manifestations differ considerably between patients.

By now, more than 2,100 genetic variants in *CFTR* have been described of which more than 400 are assumed to be pathogenic (Kounelis et al. 2020; Xiao and Lauschke 2021). Pathogenic variants are classified into five categories: variants that cause defective protein production, mostly due to premature stop codons or frameshift mutations or large insertions (class I); variants that result in defective protein trafficking (class II); variants causing defects in protein gating (class III) or dysfunctional protein conductance (class IV); and variants that cause reduced amounts of functional proteins, mostly due to splicing defects (class V).

Overall, the class II variant p.F508del (rs1801178) is most common, accounting for 70–75% of CF cases in individuals of European descent (Watson et al. 2004). In contrast, p.F508del is less common in ethnogeographic groups from Africa and Asia. Further misfolding variants include p.N1303K (rs80034486) and p.I507del (rs1490508086) that explain up to 2.8% of CF cases in Ashkenazim and up to 1.9% in Africans, respectively (Table 5). Splicing defect variants (class V) that substantially reduce the amount of functional CFTR at the plasma membrane include c.2988G > A (3120 + 1G > A), c.3717 + 12191C > T (3849 + 10kbC > T) as well as various other rare CFTR rearrangements and are of substantial relevance in Africa, where they constitute a frequent, in some groups even the most common, variant class associated with CF (Goldman et al. 2001; Macek et al. 1997; Schrijver et al. 2016; Owusu et al. 2020).

**Table 5:** *CFTR* variations and their targeted pharmacological management

Class	Description	Variant examples	CFTR quantity (% of WT)	CFTR function (% of WT)	Targeted treatment	Frequency (%)
I	Defective protein production	p.G542X	No functional protein made		Read-through agents*	4
		p.W1282X				1.8
		p.R553X				1.5
II	Trafficking defects	p.F508del	10 ± 1.7%	0.2 ± 0.2%	Ivacaftor + lumacaftor, tezacaftor and/or elexacaftor	74
		p.N1303K	3.2 ± 1.3%	0.5 ± 0%	None	2.4
		p.I507del	0 ± 0%	0.2 ± 0.1%	None	0.7
III	Gating defects	p.G551D	102.4 ± 2.9%	1.3 ± 0.4%	Ivacaftor	3.3
		p.S549N	101.9 ± 0.6%	1.6 ± 0.4%	Ivacaftor	0.2
IV	Reduced channel conductance	p.R347P	66.9 ± 2.1%	0 ± 0%	None	0.6
V	Reduced amounts of functional channels	c.2988 + 1G > A	1.3 ± 0.5%	0 ± 0%	None	0.5
		c.3717 + 12191C > T	N/A		Ivacaftor + tezacaftor	1.3

Asterisk indicates targeted treatments that are currently undergoing clinical development. “Frequency” refers to the carrier frequency among 89,052 cystic fibrosis patients registered in the CFTR2 database (https://cftr2.org/). N/A = functional in vitro data not available. Frequencies of the shown alleles in the general population extracted from gnomAD are provided in Supplementary Table 4

The major variant that causes the generation of correctly trafficked but dysfunctional channel proteins (class III) is p.G551D (rs75527207). While this variant only contributes minorly (< 1%) to cystic fibrosis risk in Hispanics and Ashkenazim, it explains between 2 and 3.5% of cases in non-Hispanic Caucasians and Asian Americans (Watson et al. 2004). Other variants resulting in CFTR dysfunction include p.R347P (rs77932196) and the Asian-specific variant p.S549N (rs121908755).

There is substantial heterogeneity within the larger populations. For instance, on average only 3–5% of European CF patients carry class III, IV or V variants; however, up to 14% of CF patients in Ireland have at least one class III variant, while more than 12% of patients in Moldova carry at least one class V mutation (De Boeck et al. 2014). Importantly, which genetic factors underlie the disease in a given patient determines the choice of pertinent therapy. Activity of reduced function CFTR proteins that have been correctly trafficked to the plasma membrane can be stimulated using “CFTR potentiators” (ivacaftor), while “CFTR correctors” (lumacaftor, tezacaftor and elexacaftor) can act as molecular chaperones to support channel folding and correct delivery of the transporter to the plasma membrane. Read-through agents (ataluren and ELX-02) have been suggested for carriers of premature termination codons that account for up to 12% of pathogenic CF alleles. However, ataluren failed to show improvement in clinical outcomes in a phase III trial and further development was hence halted (Aslam et al. 2017). ELX-02 showed promising results in vitro and phase II trials are currently ongoing (Kerem 2020). Combined, these data indicate that around 80% of CF patients in European populations carry at least one allele that renders them susceptible to treatment with currently available CFTR potentiators and CFTR correctors (p.F508del, p.G551D, p.S549N and c.3717 + 12191C > T). In contrast, the fraction of patients with suitable genotypes is considerably lower in African (∼ 60%), Hispanic (∼ 55%), Asian (∼ 45%) and Ashkenazi Jewish individuals (∼ 40%).

## *G6PD*

*G6PD* encodes glucose-6-phosphate dehydrogenase, a key enzyme in the pentose phosphate pathway that regulates NADPH levels, which is essential for redox homeostasis. Importantly, *G6PD* is highly polymorphic, and more than 200 variants have been shown to cause reduced G6PD activity (Beutler and Vulliamy 2002). While mostly asymptomatic, G6PD deficiency can be of importance upon exposure to certain triggers of oxidative stress, particularly in erythrocytes that lack mitochondria and are thus reliant on G6PD for the synthesis of redox equivalents. Triggers can be dietary components, such as fava beans or legumes, different bacterial or viral infections, or various chemically diverse drugs, such as primaquine, dapsone, sulfonamide antibiotics and rasburicase. Under these circumstances G6PD deficiency strongly increases the risk of sometimes life-threatening acute hemolytic anemia. Notably, *G6PD* is located on the X-chromosome and thus primarily impacts hemizygous males and homozygous females, whereas among heterozygous females only around 8–20% exhibit clinically relevant levels of reduced G6PD activity (Chu et al. 2018; Dechyotin et al. 2021; Johnson et al. 2009; Satyagraha et al. 2021).

G6PD deficiency is most common in Africa, followed by Southeast Asia and the Middle East (Koromina et al. 2021; Nkhoma et al. 2009). While overall disease prevalence might be similar between these regions, the genetic basis of G6PD deficiency differs drastically (Table 6). Of note, *G6PD* variant alleles are commonly referred to by their trivial names, which is a convention we will also follow in this review. In Sub-Saharan Africa, the A-^202A/376G^ allele is most common with frequencies around 10% and local peaks up to 24%, followed by A-^968C/376G^ with frequencies around 1% (Awandu et al. 2018; May et al. 2000; Pernaute-Lau et al. 2021). However, frequency profiles can be reversed in specific ethnogeographic groups, as demonstrated for West African populations in Senegal and Guinea where the A-^968C/376G^ is predominant (approximately 7–11% for A-^968C/376G^ vs. 1–3% for A-^202A/376G^) (De Araujo et al. 2006; Howes et al. 2013). Further West African alleles include the Sierra Leone (or A-^311A/376G^) variant, which however has not been extensively characterized with high geographic resolution (Jalloh et al. 2008). In contrast to Sub-Saharan Africa, the different A- alleles are very rare in East African populations (Assefa et al. 2018; Hamid et al. 2019). These results have potentially important implications for public health decisions, particularly for the treatment and prevention of malaria, as they support the roll out of primaquine, a drug associated with major anemia risk in G6PD deficient individuals, as radical cure for *Plasmodium vivax* and as transmission interruption for *Plasmodium falciparum* in East Africa, whereas *G6PD* genotyping before the initiation of 8-aminoquinolone therapy is warranted in South and West Africa. However, the status of other deficient variants beyond A- should be evaluated in East Africa to further corroborate this conclusion.

**Table 6:** *G6PD* alleles of major clinical relevance and their ethnogeographic distribution

Allele	Variants	Protein effect	Functional consequence	Main ethnogeographic groups (MAF in the general population)	References
Mediterranean	rs5030868	p.S188F	Severe	Middle East and Southcentral Asia (1–9%)	Al-Allawi et al. (2010), Alfadhli et al. (2005), Doss et al. (2016), Jamornthanyawat et al. (2014)
Canton	rs72554665	p.R459L	Severe	North Vietnam and South China (up to 6%)	He et al. (2020), Sathupak et al. (2021), Zheng et al. (2020)
Kaiping	rs72554664	p.R463H	Severe	North Vietnam and South China (up to 5%)	He et al. (2020), Sathupak et al. (2021), Zheng et al. (2020)
Viangchan	rs137852327	p.V291M	Severe	Southeast Asia, mostly Laos, Cambodia, Vietnam and Malaysia (up to 6%)	Matsuoka et al. (2005)
Chatham	rs5030869	p.A335T	Severe	Central Asia (up to 3% in Iran)	Al-Allawi et al. (2010), Mesbah-Namin et al. (2002), Karimi et al. (2003)
Vanua Lava	rs78365220	p.L128P	Severe	Pacific islands (up to 5%)	Ganczakowski et al. (1995), Satyagraha et al. (2015)
A-^202A/376G^	rs1050828 and rs1050829	p.V68M and p.N126D	Moderate	Central Sub-Saharan Africa (10–24%)	Awandu et al. (2018), May et al. (2000), Pernaute-Lau et al. (2021)
A-^968C/376G^	rs1050829 and rs76723693	p.N126D and p.L323P	Moderate	West Africa (up to 11%)	De Araujo et al. (2006), Howes et al. (2013)
Cairo	rs782322505	p.N135T	Moderate	Middle East (up to 0.4%)	Koromina et al. (2021)
Kalyan-Kerala	rs137852339	p.E317K	Moderate	India (3%)	Chalvam et al. (2007), Devendra et al. (2020)
Orissa	rs78478128	p.A44G	Moderate	India (1–3%)	Devendra et al. (2020)
Mahidol	rs137852314	p.G163S	Moderate	Southeast Asia, mostly Myanmar, Thailand and Burma (2–6%)	Matsuoka et al. (2004), Phompradit et al. (2011)

Severe deficiency indicates < 10% residual enzyme activity while moderate deficiency refers to enzyme activities between 10 and 60%. MAF = minor allele frequency. For a detailed overview of the frequencies of the indicated alleles in different ethnogeographic groups, we refer to a recent population-scale analysis (Koromina et al. 2021)

In Middle Eastern populations G6PD deficiency is primarily attributed to the Mediterranean allele (Doss et al. 2016), accounting, for instance, for 88% and 74% of G6PD deficiency among the Kurdish population in Northern Iraq and in Kuwaiti Arabs (MAF in the general population = 1–4%), respectively (Al-Allawi et al. 2010; Alfadhli et al. 2005). Further relevant G6PD deficient variants in the Middle East are A-^968C/376G^, Cairo and Chatham, with overall MAFs of 0.4–0.8%. The Mediterranean variant is furthermore common in Southcentral Asia with frequencies up to 8.9% in Afghani Pashtun (Jamornthanyawat et al. 2014). While it also constitutes a relevant factor in India, explaining around 24% of G6PD deficiencies in a country-wide survey, the overall most prevalent allele was Orissa, which accounted for 57% of all deficiencies (Devendra et al. 2020). Further rare variants of relevance in specific South Asian subpopulations and tribal groups are Kalyan–Kerala and Namoru (Chalvam et al. 2007). In Southeast Asia, the predominant allele is Mahidol, which explains 38–96% in of G6PD deficiencies in Burma, Thailand and Myanmar (Matsuoka et al. 2004; Phompradit et al. 2011). In contrast, G6PD deficiency in Cambodia was almost exclusively caused by the Viangchan allele (Matsuoka et al. 2005). Furthermore, specific subpopulations feature unique molecular G6PD patterns; for instance, the otherwise rare Aures allele constitutes the most common G6PD deficient variant in the Lao Theung population, the second largest ethnic group in Laos (Sanephonasa et al. 2021).

Compared to the variant profile in South and Southeast Asian populations, *G6PD* variability in China is distinctly different. In Han Chinese, Kaiping (MAF = 0.3%) and Canton (MAF = 0.3%) were the most common *G6PD* deficient alleles and showed a clear South-to-North national gradient (He et al. 2020). In other Chinese ethnic groups, such as Dai, Miao, Tibetans and Yi, variant signatures showed pronounced differences with the *G6PD* Gaohe, Baise, Fushan and Union alleles explaining > 10% of population-specific deficiencies (Zheng et al. 2020). In contrast to China where the country-wide prevalence of G6PD deficiency is around 1.9% among males, G6PD deficiency is a rare disorder in Japan with an overall frequency of < 0.1%. Notably, despite this low frequency, a multitude of distinct very rare Japanese deficient alleles have been described, including Fukushima, Morioka, Yamaguchi and Musashino. Combined, these results demonstrate the conspicuous differences in *G6PD* molecular genetics even across ethnic groups in close geographical proximity and indicate that it is essential to employ genotyping strategies that are tailored to the specific population or ethnic background of a given patient.

## Opportunities for precision public health

Population pharmacogenomic profiling can reveal genetic differences that predispose to differences in drug response. In Europeans, reduced function alleles of *CYP2D6* are considerably more frequent than in other populations. Thus, genetic testing is particularly beneficial in these populations for identifying outlier patients, such as CYP2D6 poor metabolizers. The respective information can be utilized clinically, e.g. for prescribing alternatives to tramadol and codeine analgesics for pain relief (Crews et al. 2021) and for recommending aromatase inhibitors, such as anastrozole instead of tamoxifen for the prevention of breast cancer recurrence (Goetz et al. 2018)). Furthermore, European populations harbour the highest frequencies of CFTR trafficking mutations, suggesting that the rate of cystic fibrosis patients responding to CFTR correctors is overall higher in Europe compared to other populations.

Reduced function variants of *DPYD* and *TPMT* are most prevalent in Sub-Saharan Africa and, thus, preemptive genetic testing and genotype-guided dose adjustments of fluoropyrimidines and thiopurines are particularly beneficial in those populations. Similarly, African populations have the highest frequencies of genetic *G6PD* deficiency, which constitutes a contraindication to treatment with the 8-aminoquinoline antimalarials primaquine and tafenoquine, the only curative treatments for *Plasmodium vivax* malaria, due to drastically elevated risk of severe acute haemolytic anaemia (Watson et al. 2018). Furthermore, G6PD deficiency status is useful to guide treatment with various other drugs, including pegloticase, rasburicase, flutamide, as well as sulfonamide antibiotics.

Southeast Asia constitutes the main hotspot of the HLA-B*15:02 and HLA-B*58:01 alleles, entailing that testing for carbamazepine and allopurinol induced severe cutaneous adverse reactions is most important in these groups. Notably, country-specific frequency information can refine pharmacogenomic decision making at the national level. For example, while HLA-B*15:02 is generally common in Asian populations with average minor allele frequencies of 5–10%, rates are much higher in the Philippines where about half of the population are carriers, whereas frequencies in Japan are < 0.1%. With increasing availability of genotype information, genetic differences between ethnic groups are revealed with higher and higher resolution and the resulting data shows that pronounced genetic differences can exist even across relatively small geographic regions. However, we want to emphasize that both high resolution studies with well-defined cohorts as well as population-scale aggregated information should be considered to allow for an integration of information about ethnogeographic differences with modern human migration and admixture.

## Conclusions

Interindividual differences in drug response are in part caused by genetic variants with differential ethnogeographic prevalence and information about their distribution can be important for population-stratified therapy (Mette et al. 2012; Roberts et al. 2021; Yasuda et al. 2008). In this review, we provide a current update of population differences in the genetic variability of ten different genes that are included in the labels of 141 different drugs or therapeutic regimens as warnings or as factors impacting the clinical pharmacology of the agents in question (Supplementary Table 5). The aggregated data suggest strong differences in variant distribution and gene functionality between major ethnogeographic groups. We hope that the overview provided herein can serve as a useful resource for pharmacologists, clinical geneticists and public health researchers to evaluate treatment risks and inform population-adjusted genotyping strategies.

## Supplementary Information

Below is the link to the electronic supplementary material.

Supplementary Table 1: References for population-specific frequencies of *CYP2D6* alleles. PMIDs or, if the paper was not indexed in Pubmed, DOIs are provided (XLSX 13 KB)

Supplementary Table 2: References for population-specific frequencies of CYP2C19 alleles. PMIDs or, if the paper was not indexed in Pubmed, DOIs are provided (XLSX 11 KB)

Supplementary Table 3: References for population-specific frequencies of DPYD alleles. PMIDs or database sources are provided (XLSX 9 KB)

Supplementary Table 4: Population-specific frequencies of the most common functionally deficient CFTR alleles in the general population. Data is extracted from 141,456 individuals provided by gnomAD v2.1.2 (https://gnomad.broadinstitute.org/) (XLSX 10 KB)

Supplementary Table 5: Drugs and regimens for which the 10 analyzed genes impact the clinical pharmacology, efficacy or safety. Information obtained from https://www.fda.gov/drugs/science-and-research-drugs/table-pharmacogenomic-biomarkers-drug-labeling [Accessed 08.08.2021] (XLSX 13 KB)
